# Neurobehavioral phenotype of Kabuki syndrome: Anxiety is a common feature

**DOI:** 10.3389/fgene.2022.1007046

**Published:** 2022-10-06

**Authors:** Allison J. Kalinousky, Tyler Rapp, Hadia Hijazi, Jennifer Johnson, Hans Tomas Bjornsson, Jacqueline R. Harris

**Affiliations:** ^1^ McKusick-Nathans Department of Genetic Medicine, Johns Hopkins University School of Medicine, Baltimore, MD, United States; ^2^ University of North Carolina School of Medicine, University of North Carolina, Chapel Hill, NC, United States; ^3^ Kennedy Krieger Institute, Baltimore, MD, United States; ^4^ Faculty of Medicine, University of Iceland, Reykjavík, Iceland; ^5^ Landspitali University Hospital, Reykjavík, Iceland

**Keywords:** epigenetics, KMT2D (MLL2), mendelian disease, neurodevelopment disorder, KDM6A

## Abstract

Kabuki syndrome (KS) is a Mendelian Disorder of the Epigenetic Machinery (MDEM) caused by loss of function variants in either of two genes involved in the regulation of histone methylation, *KMT2D* (34–76%) or *KDM6A* (9–13%). Previously, representative neurobehavioral deficits of KS were recapitulated in a mouse model, emphasizing the role of KMT2D in brain development, specifically in ongoing hippocampal neurogenesis in the granule cell layer of the dentate gyrus. Interestingly, anxiety, a phenotype that has a known association with decreased hippocampal neurogenesis, has been anecdotally reported in individuals with KS. In this study, anxiety and behavior were assessed in a cohort of 60 individuals with molecularly confirmed KS and 25 unaffected biological siblings, *via* questionnaires (SCARED/GAS-ID and CBCL/ABCL). Participant age ranged from 4 to 43 years old, with 88.3% of participants having a pathogenic variant in *KMT2D*, and the rest having variants in *KDM6A*. In addition, data was collected on adaptive function and positive affect/quality of life in participants with KS using appropriate online surveys including ABAS-III and PROMIS Positive Affect. Survey scores were compared within the KS participants across age groups and between KS participants and their unaffected siblings. We found that children with KS have significantly higher anxiety scores and total behavior problem scores than their unaffected siblings (*p* = 0.0225, *p* < 0.0001). Moreover, a large proportion of affected individuals (22.2% of children and 60.0% of adults) surpassed the established threshold for anxiety; this may even be an underestimate given many patients are already treated for anxiety. In this sample, anxiety levels did not correlate with level of cognitive or adaptive function in any KS participants, but negatively correlated with positive affect in children with KS (*p* = 0.0005). These findings indicate that anxiety is a common neurobehavioral feature of KS. Providers should therefore carefully screen individuals with KS for anxiety as well as other behavioral issues in order to allow for prompt intervention. Neurobehavioral anxiety measures may also prove to be important outcome measures for clinical trials in KS.

## Introduction

Kabuki syndrome (KS; Niikawa-Kuroki syndrome) is a Mendelian Disorder of the Epigenetic Machinery (MDEM) characterized by intellectual disability, postnatal growth deficiency, dysmorphic facial features, persistent fetal fingertip pads, and skeletal anomalies (Niikawa et al., 1988). KS can be diagnosed clinically or on the basis of a pathogenic variant in one of two genes, *KMT2D* (MIM#147920) resulting in KS type 1, or *KDM6A* (MIM#300867) resulting in KS type 2 (Adam et al., 2019). The majority (34–76%) of molecularly confirmed cases possess variants in *KMT2D*, which encodes a histone methyltransferase that adds mono- and tri-methyl groups to the fourth lysine (K4) of histone 3, promoting open chromatin (Van Laarhoven et al., 2015; Harris et al., 2019). A smaller subset (9–13%) of individuals with KS have variants in *KDM6A*. KDM6A is a histone demethylase that removes H3K27me3, a closed chromatin mark, also therefore promoting open chromatin (Cheon et al., 2014).

School-aged children and adults with KS have a very specific cognitive profile characterized by intellectual disability (ID)—typically in the mild range—with visuospatial construction, perception, and memory far more impaired than other areas of cognition, while language ability is relatively spared (Harris et al., 2019). Previously, representative neurobehavioral deficits were recapitulated in a mouse model of KS, emphasizing the role of KMT2D in brain development and function, specifically in hippocampal neurogenesis in the granule layer of the dentate gyrus (Bjornsson et al., 2014). In combination, these findings provide evidence that at least some of the main neurological deficits in KS localize to the dentate gyrus, and to deficient neurogenesis in that region. While the cognitive profile associated with KS has been investigated, less is known about other neurodevelopmental and neurobehavioral anomalies that are often observed in affected individuals. For example, previous studies have noted the presence of deficits such as adaptive skill impairment, autistic-like behavior, psychiatric pathologies, and impaired emotional control (Boniel et al., 2021), however, this was based mainly on qualitative, non-systematic reporting, and to our knowledge, no studies have specifically looked at these areas in depth. Interestingly, anxiety, an anecdotally reported trait in individuals with KS, is also an understudied neurobehavioral feature of KS, despite being a well-known phenotype associated with decreased hippocampal neurogenesis (Kheirbek and Hen, 2014). It is estimated that 3–22% of individuals with ID have anxiety (Reardon et al., 2015) and those with genetic syndromes causing their ID have even more elevated rates of anxiety disorders. For example, both individuals with Williams syndrome, a genetic syndrome characterized by mild to moderate ID, and individuals with Fragile X syndrome, a genetic disorder that causes ID, have been found to have a rate of anxiety of approximately 48% (Royston et al., 2016; Edwards et al., 2022). CHARGE syndrome, a syndrome that is known to have phenotypic overlap with KS, has been reported as having an anxiety prevalence of 37% (Schulz et al., 2014; Edwards et al., 2022). As these and some genetic syndromes with ID have much higher rates of anxiety disorders than individuals with idiopathic or non-specific ID, it stands to reason that specific effects of the molecular underpinnings of these syndromes cause the anxiety, beyond just the presence of intellectual disability.

While no specific therapy exists for KS, the treatment of some of the specific neurobehavioral aspects of KS, such as anxiety, is possible and can help in ameliorating the overall severity of symptoms (Mendlowicz and Stein, 2000). Additionally, anxiety has been previously reported to be one of the issues substantially impacting caregiver and patient burden (Theodore-Oklota et al., 2021). Beyond just treating the anxiety itself, preclinical studies in MDEMs in general, and in KS specifically, have shown that some neurological and functional deficits can be rescued postnatally (Alarcón et al., 2004; Korzus et al., 2004; Bjornsson et al., 2014; Benjamin et al., 2017). It is therefore crucial that we understand the neurodevelopmental and neurobehavioral profiles of KS in depth in order to design outcome measures for targeted treatments and provide optimal clinical care to individuals with KS.

Based on previously published evidence of hippocampal neurogenesis deficits in the KS mouse model (Bjornsson et al., 2014) and the anecdotal evidence seen by clinicians, we hypothesized that individuals with KS would have significantly higher levels of anxiety than their biological siblings without KS living in the same household. To investigate these hypotheses, we administered a set of parent-reported and adult self-reported questionnaires to further understand anxiety, behavior, and adaptive function in individuals with KS. We then compared scores acquired from affected individuals to their unaffected siblings to understand the specificity of this feature to KS.

## Materials and methods

### Participants

This study was approved by the Johns Hopkins Institutional Review Board and written informed consents were collected from all participants or legal guardians prior to study recruitment and participation. The study population consisted of individuals with a molecularly confirmed diagnosis of KS type 1 or 2 recruited from the Epigenetics and Chromatin clinic at Johns Hopkins or the Epigenetics Clinic at the Kennedy Krieger Institute, as well as from participants who previously consented to be contacted about relevant research studies. Individuals aged 4 years or older at the time of recruitment were included. Genetic variants of all participants are pathogenic or likely pathogenic by ACMG-AMP criteria, except for two variants of uncertain significance that were subsequently confirmed to cause KS by Episign Variant testing (Aref-Eshghi et al., 2017). In addition to genetic testing reports, historical clinical neuropsychology reports were collected from all participants, when possible. Use of anxiety medication was reported by parents/guardians of all KS participants. Behavioral data on healthy biological siblings of child participants with KS who lived in the same household was collected to use as control data. Siblings were used as the control group in an attempt to isolate anxiety solely due to KS and not due to familial predisposition or environmental factors that could contribute to the development of anxiety. Demographics of participants can be found in Tables 1 and 2, and in Supplementary Table 1.

**TABLE 1:** Demographics of study participants with KS.

	All participants (*n* = 60)	Children (*n* = 45)	Adults (*n* = 15)	*KMT2D* (*n* = 53)	*KDM6A* (*n* = 7)
*Sex*					
*Male*	29	24	5	29	0
*Female*	31	21	10	24	7
*On anxiety meds*	20	14	6	18	2
*Age (average, range)*	13.93Y (4-44Y)	10.20Y	25.13Y	14.38Y (4-43Y)	10.57Y (8-19Y)
*General cognitive function (average, range)*	73.88 (47–94)	72.33 (56–94)	75.70 (47–94)	75.38 (56–94)	56 (47–65)

**TABLE 2:** Demographics of unaffected biological siblings of participants with KS.

ID	Age	Sex	Sibling age	Sibling sex
*KS0221*	10	M	6	F
*KS0722*	11	F	8	M
*KS1121*	16	F	15	F
*KS1821*	5	M	7	F
*KS2121*	5	M	8	M
*KS2421*	5	F	10	F
*KS2821*	7	F	12	M
*KS3221*	13	M	16	F
*KS3321*	9	F	7	M
*KS3421*	5	F	3	F
*KS3622*	7	F	11	M
*KS3921*	7	F	9	F
*KS4122*	13	M	10	M
*KS4621*	10	F	14	M
*KS5221*	8	F	5	F
*KS5422*	16	M	20	M
*KS5622*	6	F	4	M
*KS5721*	13	M	11	M
*KS6121*	5	M	15	F
*KS6322*	5	M	10	M
*KS6822*	4	M	13	F
*KS6921*	11	F	8	M
*KS7121*	7	F	9	F
*KS7322*	11	M	16	F
*KS7522*	11	F	10	M

### Instrumentation

Parents or guardians of individuals with KS under the age of 18 electronically completed the Screen for Child Anxiety Related Emotional Disorders (SCARED), a questionnaire designed as a screening tool to use in a clinical setting for pediatric anxiety disorders and has been validated in ID populations (Lohr et al., 2017; Robles-Bello et al., 2020). The informant answers each item as not true or hardly ever true, somewhat true or sometimes true, or very true or often true, which is then rated as 0, 1, or 2, respectively. This is summed together to provide an overall score for anxiety, with a maximum score of 82, and a score of 25 or higher potentially indicating an anxiety disorder. The SCARED questionnaire also provides five subscale scores: panic/somatic anxiety, generalized anxiety, separation anxiety, social phobia, and school phobia. For each of the sub-categories, there are certain thresholds that if met, indicate that the individual could have that specific type of anxiety disorder (Birmaher et al., 1997). If the child with KS had a sibling living in the same household, the same respondent also completed a SCARED survey for the unaffected biological sibling.

Individuals with KS over the age of 18 electronically self-completed the Glasgow Anxiety Scale for people with an Intellectual Disability (GAS-ID), which was designed to assess state anxiety in people with mild intellectual disability. The individual responds never, sometimes, or always to each item, which corresponds to 0, 1, or 2, respectively. The maximum score is 54, with a score of 13 or higher potentially indicating an anxiety disorder (Mindham and Espie, 2003).

Additionally, age appropriate versions of the Adaptive Behavior Assessment System-Third Edition (ABAS-III) (Oakland, 2011), the Child Behavior Checklist (CBCL) or the Adult Behavior Checklist (ABCL) (Achenbach and Rescorla, 2001), and the proxy version of the Patient Reported Outcome Measurement Information System (HealthMeasures, 2018)—positive affect scale (Forrest et al., 2017) were distributed electronically to the individual with KS, if over the age of 18, or to their parent/guardian, if under the age of 18, for reporting. The ABAS-3 scores an individual’s general adaptive behavior, focusing on 10 adaptive skill areas: communication, community use, functional academics, school/home living, health and safety, leisure, self-care, self-direction, social, and work (if applicable). The ABAS-3 generates standard scores with a mean of 100 and a standard deviation of 15 (Oakland, 2011). Behavior checklists from the Achenbach System of Empirically Based Assessment were used to assess behavioral and emotional problems, scaling for internalizing, externalizing, and total problems. This was distributed to investigate if other behavioral problems are present in individuals with KS. Responses are given *via* Likert-scale and reports are normed by sex and age band with those beyond the 97th percentile being clinically significant (Achenbach and Rescorla, 2001). The parent-proxy edition of the PROMIS Pediatric Positive Affect was used for those under of the age of 18. The parent-proxy edition was created for use for children who are too young, cognitively impaired, or too ill to complete the patient-reported version of this assessment (*POSITIVE AFFECT A brief guide to the PROMIS® Positive Affect instruments.*). The item bank of this assessment uses a 7-day recall period covering six content categories, including low activation states of contentment, love, and pride, and high activation states of happiness, excitement, and energy in order to assess the individual’s level of happiness and positive affect (Forrest et al., 2017). The adults completed a self-reported health-related quality of life measure.

### Statistical analysis

Data distributions were examined for normality using the Shapiro-Wilk test (Shapiro and Wilk, 1965). Parametric tests were used for normally distributed data, while non-parametric tests were used for non-normally distributed data. All correlation tests were performed in RStudio and a *p*-value of 0.05 or lower was considered significant (RStudio Team, 2019). Two-tailed paired t-tests were performed when comparing scores of individuals with KS to their siblings, while two-tailed unpaired t-tests were performed when comparing differences between groups (i.e. KS1 *versus* KS2 and truncating *versus* missense variants in *KMT2D*). The t-tests were all preformed using GraphPad Prism version 9.3.1 for Mac OS X, GraphPad Software, La Jolla California United States, www.graphpad.com. Given the exploratory and descriptive nature of this study, no corrections were made for multiple comparisons. Further studies are required before using any of this data for clinical trial design, so at this stage the risk of a type I error is not very consequential.

## Results

### Clinical and molecular characteristics of participants

**FIGURE 1:**
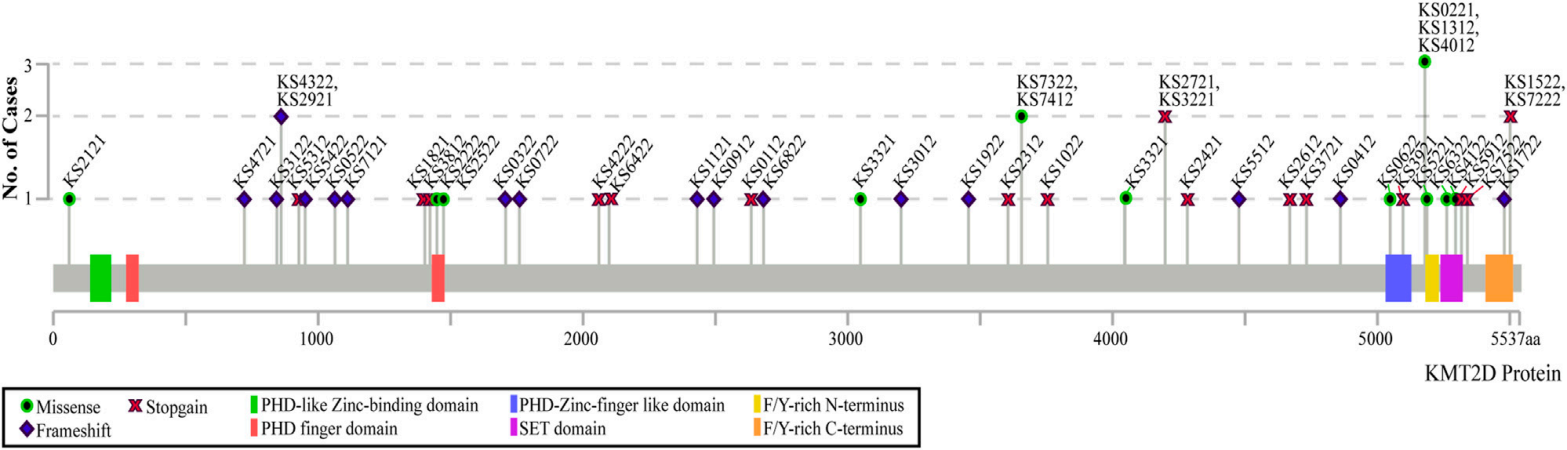
Distribution of participants’ variants across the *KMT2D* protein domains. The type of variant is indicated by the symbol on the graph, with the height of each point representing the number of individuals with that specific variant. Only coding variants of the *KMT2D* gene are shown. All coordinates are in genome build hg38.

Sixty individuals with KS were included in the study, 45 of which had a parent/guardian complete parent-proxy surveys for children and 15 of which self-completed the adult surveys. The ages of the participants with KS ranged from 4 to 43 years, with the mean age of the children group being 10.20Y (SD 4.66Y), and the mean adult group age being 25.13Y (SD 6.75Y). Twenty-four of the 45 children (53.33%) and five of the 15 adults (33.33%) are males, while the rest are females. Seven of the 60 recruited individuals have variants in *KDM6A*, with the remaining having variants in *KMT2D*. Thirty-seven individuals have truncating variants and 13 individuals have missense variants in *KMT2D*. Figure 1 shows the distributions of variants throughout the *KMT2D* gene, created in MutationMapper (Cerami et al., 2012; Gao et al., 2013). Fifteen of the 45 children provided historical neuropsychology reports that reported some measure of general cognitive function as measured by full-scale intelligence quotient (FSIQ), or when FSIQ was not available, by fluid crystallized index (measured by Kaufman Assessment Battery for Children), or general ability index (measured by Wechsler Intelligence Scales for Children-V (WISC-V)). All of these scores have a population mean of 100 and a standard deviation of 15. The average general cognitive function score for children with KS in this study who reported this information is 72.33 (SD 11.10). Ten of the 15 adults provided neuropsychological testing with reported FSIQ scores. The average FSIQ score for the adults in this study who reported this information is 75.70 (SD 14.16). Table 1 shows the demographics of all the participants with KS. Twenty-five siblings were included in this study, with ages ranging from 3 to 20 years, with the mean age being 10.28Y (SD 4.04Y). Table 2 shows the demographics of the unaffected siblings and demographics of participants with KS that have siblings. Detailed clinical and molecular information is included in Supplementary Table S1.

### Prevalence of anxiety in individuals with Kabuki syndrome

**FIGURE 2:**
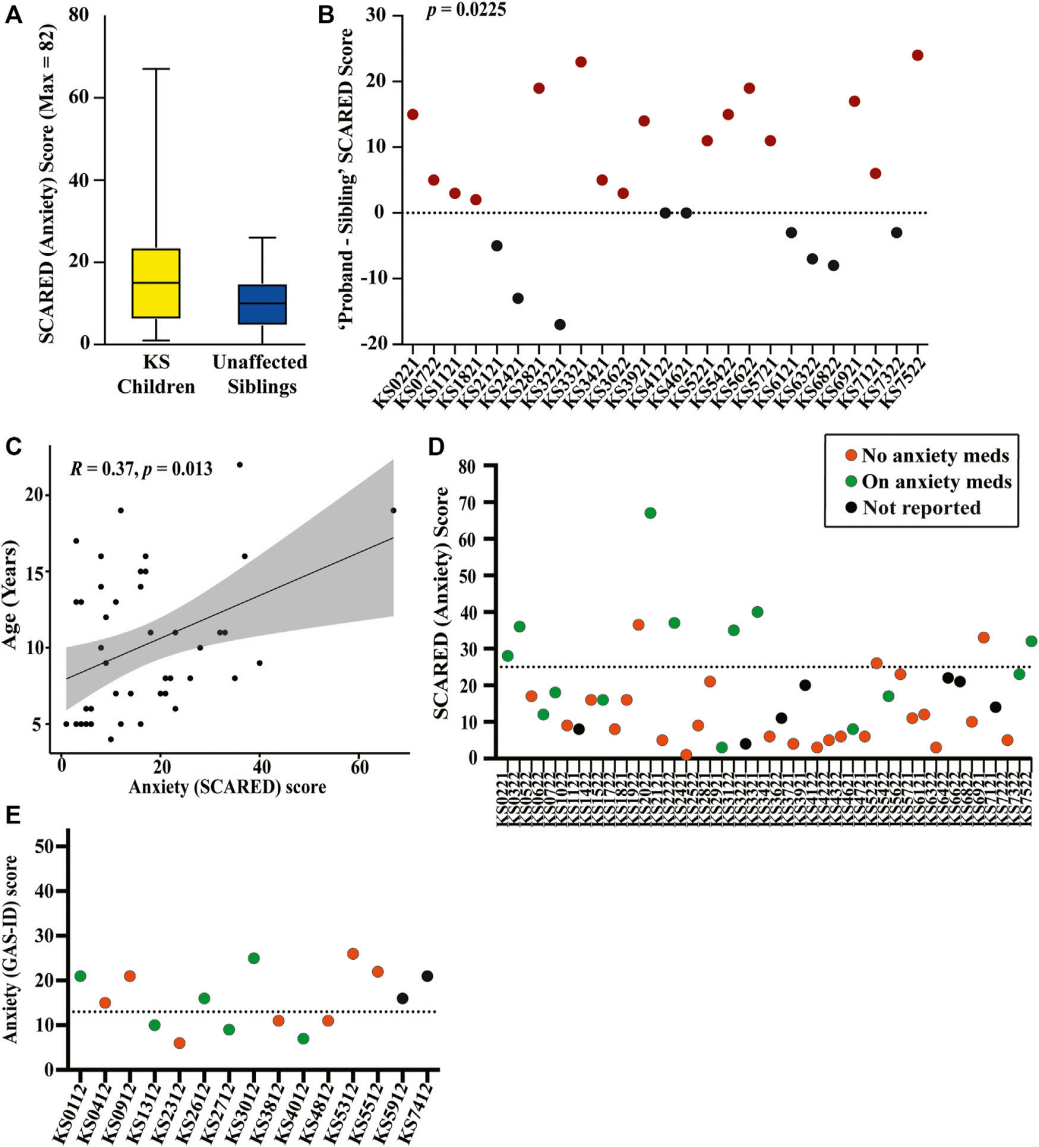
Graphical representation of results from the collected SCARED and GAS-ID anxiety surveys **(A)** SCARED survey scores for children with KS, shown in yellow, and unaffected siblings (when available) as a control group, shown in blue. **(B)** The difference in SCARED scores between each KS proband (ID displayed on the *x*-axis) and their unaffected sibling. Paired-comparison confirms there being a significant difference in the score of affected *versus* unaffected child within sibling pairs (*p* = 0.0225). **(C)** Spearman’s rank correlation plot between age and anxiety severity (score) for children with KS (*p* = 0.0135). **(D)** Plot showing children with KS (ID displayed on the *x*-axis) above and below the SCARED threshold for anxiety. Those who are actively taking medication for anxiety are shown in green, those that are not are shown in orange, and those that did not respond are shown in black. **(E)** GAS-ID scores for all adults with KS (ID displayed on the *x*-axis), with those above the dotted line representing the threshold for anxiety. Those who are actively taking medication for anxiety are shown in green, those that are not are shown in orange, and those that did not respond are shown in black.

The average SCARED score in the KS group is 16.98 (SD 13.22), with 10 of the 45 children with KS (22.22%) scoring above the threshold for anxiety. Twenty-five of the children had siblings who also completed the SCARED survey. The average score for the siblings is 10.28 (SD 6.56), with only one (4.0%) of the siblings scoring above the anxiety threshold (Figure 2A). Comparing anxiety scores directly between sibling pairs, children with KS have significantly higher scores than their unaffected sibling, with a *p*-value of 0.0225 (Figure 2B). There is a positive correlation between anxiety levels in children with KS and age (*p* = 0.0135) (Figure 2C). Of the 10 children with KS that scored above the threshold for anxiety, seven of them are currently taking anxiety medication, and three are not. Of the 35 children that scored below the threshold, seven are currently taking medication to treat anxiety, 21 are not, and seven did not report (Figure 2D). Adding together those who scored above the threshold on the full SCARED and those who scored below the threshold but who are actively receiving treatment for anxiety, 17 of the 45 children with KS (37.78%) have anxiety.

Nineteen children with KS exceeded threshold scores on one or more of the five SCARED subcategories. The most common subtype of anxiety is separation anxiety, with 13 of the children with KS (28.89%) scoring above the threshold on these questions. Ten children (22.22%) scored above the threshold on questions corresponding to social anxiety, nine children (20.0%) scored above the threshold on questions relating to significant school avoidance, eight children (17.78%) scored above the threshold on questions corresponding to a generalized anxiety disorder, and seven children (15.56%) scored above the threshold on questions relating to a panic disorder or significant somatic symptoms. Of the siblings, seven out of the 25 individuals reached one of these thresholds, with six (24.0%) scoring above the threshold on the sections indicating separation anxiety and the remaining one (4.0%) scoring above the threshold on the section corresponding to a generalized anxiety disorder (Supplementary Table S2).

The average score on the GAS-ID for the adults in this study is 15.80 (SD 6.37), with nine of the 15 adults (60.0%) scoring above the anxiety threshold. Of the nine adults that surpassed the anxiety threshold, three are currently taking anxiety medication, four are not, and two did not report. Of the six adults that score below the anxiety threshold, three are actively taking medication to treat anxiety while the remaining three are not (Figure 2E). Adding together those who scored above the threshold on the full GAS-ID and those who scored below the threshold but who are actively receiving treatment for anxiety, 12 of the 15 adults with KS (80%) have anxiety.

### Comparing molecular characteristics to anxiety

To assess whether anxiety is equally prevalent in KS type 1 compared to KS type 2, we performed an unpaired *t*-test between anxiety scores of children with KS1 compared to children with KS2. There is no significant difference in anxiety scores between the children with KS1 and KS2, with a *p*-value of 0.3373. Anxiety scores for adults were not compared as there was only one adult with KS2.

Anxiety scores from children possessing truncating variants in *KMT2D* were compared to those with missense variants in this gene to those with splice site variants in this gene. No significant difference in anxiety scores (*p* = 0.5718) was found between the three compared groups. When comparing adults possessing truncating variants in *KMT2D* to those with missense variants in this gene, there is no significant difference in anxiety scores (*p* = 0.3571). Table 3 displays averages for all assessments completed by adults and children with KS.

**TABLE 3:** Average scores on assessments completed by all individuals with KS.

Assessment		Mean (SD)	Range of possible scores	Clinically significant cutoff score	% Clinically significant
*SCARED/GAS-ID*	All children	16.98 (13.22)	0—82	≥25	22.22
	All adults	15.80 (6.37)	0—54	≥13	60.00
*CBCL/ABCL*	Internalizing problems	58.09 (11.33)	20—100	≥64	27.27
	Externalizing problems	54.84 (10.74)	20—100	≥64	21.82
	Total problems	61.84 (10.97)	20—100	≥64	47.27
*ABAS-III*	General adaptive composite	71.83 (15.41)	40—130	<90	86.44
	Conceptual	74.56 (15.27)	40—130	<90	88.13
	Practical	68.71 (15.80)	40—130	<90	88.13
	Social	81.71 (14.02)	40—130	<90	74.58
*Positive Affect/QOL*	Pediatric Parent-Proxy Positive Affect	51.8 (16.1)	10—80	NA	NA
	Adult Quality of Life	52.3 (15.0)	25—75	NA	NA

SCARED, screen for child anxiety related emotional disorders; GAS-ID, glasgow anxiety scale for people with an intellectual disability; CBCL, child behavior checklist; ABCL, adult behavior checklist; ABAS-III, Adaptive Behavior Assessment System-Third Edition; QOL, quality of life.

### General behavioral problems in Kabuki syndrome

The T-scores for internalizing problems for individuals with KS on the CBCL or ABCL ranged from 34 to 85 (mean 58.09, SD 11.33). Of these individuals, 54.55% scored in the normal range, 18.18% scored in the borderline clinical range, and 27.27% scored in the clinical range for internalizing problems. The T-scores for internalizing problems for unaffected siblings ranged from 33 to 69 (mean 48.13, SD 10.60). Of the siblings, 86.96% scored in the normal range, 0 scored in the borderline clinical range, and 13.04% score in the clinical range for internalizing problems. The T-scores for externalizing problems for individuals with KS ranged from 34 to 79 (mean 54.84, SD 10.74). Of the individuals with KS**,** 67.27% scored in the normal range, 10.91% scored in the borderline clinical range, and 21.82% scored in the clinical range for externalizing problems. The T-scores for externalizing problems for unaffected siblings ranged from 33 to 66 (mean 45.87, SD 9.77). Of the siblings, 86.96% scored in the normal range, 4.35% scored in the borderline clinical range, and 8.70% scored in the clinical range for externalizing problems. The T-scores for total problems for individuals with KS ranged from 30 to 88 (mean 61.84, SD 10.97). Of the individuals with KS, 41.82% scored in the normal range, 10.91% scored in the borderline clinical range, and 47.27% scored in the clinical range for total problems. The T-scores for total problems for unaffected siblings ranged from 25 to 65 (mean 44.48, SD 10.82). Of the siblings, 86.96% scored in the normal range, 4.35% scored in the borderline clinical range, and 8.70% scored in the clinical range for total problems. Table 4 displays averages for all behavioral categories as well as anxiety results for children with KS and the sibling group.

**TABLE 4:** Averages and *p*-values for all assessments completed by KS probands (children) and their siblings.

Assessment		KS, pooled, mean (SD)	Sibling mean (SD)	*p*-value (paired between KS probands and their siblings)^a^	% KS clinically significant	% Siblings clinically significant
*SCARED*		16.98 (13.22)	10.28 (6.56)	0.0225	22.22	4.00
*CBCL*	Internalizing problems	58.12 (10.39)	48.13 (10.60)	0.0009	26.83	13.04
	Externalizing problems	55.83 (10.16)	45.70 (9.77)	0.0011	21.95	8.70
	Total problems	62.68 (9.87)	44.48 (10.82)	<0.0001	51.22	8.70
	*Syndrome scale scores*					
	Anxious/depressed	57.02 (8.75)	53.96 (5.64)	0.1394	9.76	0
	Somatic Complaints	60.85 (8.68)	54.52 (6.32)	0.0005	19.51	0
	Withdrawn/depressed	58.41 (8.22)	52.70 (4.65)	0.0230	7.32	0
	Social problems	64.98 (6.22)	51.61 (2.97)	<0.0001	19.51	0
	Thought problems	64.07 (12.85)	52.74 (4.84)	0.0008	39.02	0
	Attention problems	70.15 (10.54)	52.26 (4.24)	<0.0001	48.78	0
	Rule-breaking behavior	55.44 (5.85)	52.30 (3.76)	0.01443	2.44	0
	Aggressive behavior	58.95 (10.05)	53.17 (5.73)	0.0052	19.51	0
	*DSM-oriented scale*					
	Depressive problems	61.63 (8.46)	53.09 (6.06)	0.0004	14.63	4.35
	Anxiety problems	58.95 (12.82)	53.61 (4.92)	0.0305	14.63	0
	Somatic problems	59.30 (8.57)	53.74 (5.71)	0.0038	14.63	0
	Attention deficit	65.85 (8.99)	51.96 (3.98)	<0.0001	36.59	0
	Oppositional defiant problems	57.85 (8.54)	53.91 (6.50)	0.0381	14.63	4.35
	Conduct problems	56.41 (7.91)	52.30 (4.55)	0.0142	7.32	0
	Sluggish cognitive tempo	62.90 (8.76)	51.52 (3.68)	0.0003	31.71	0
	Obsessive-compulsive problems	63.46 (9.84)	54.00 (4.38)	0.0001	26.83	0
	Stress problems	62.34 (8.15)	52.61 (4.40)	0.0001	14.63	0

a paired *t*-test.

SCARED, screen for child anxiety related emotional disorders; CBCL, child behavior checklist; pooled includes all children with KS.

**FIGURE 3:**
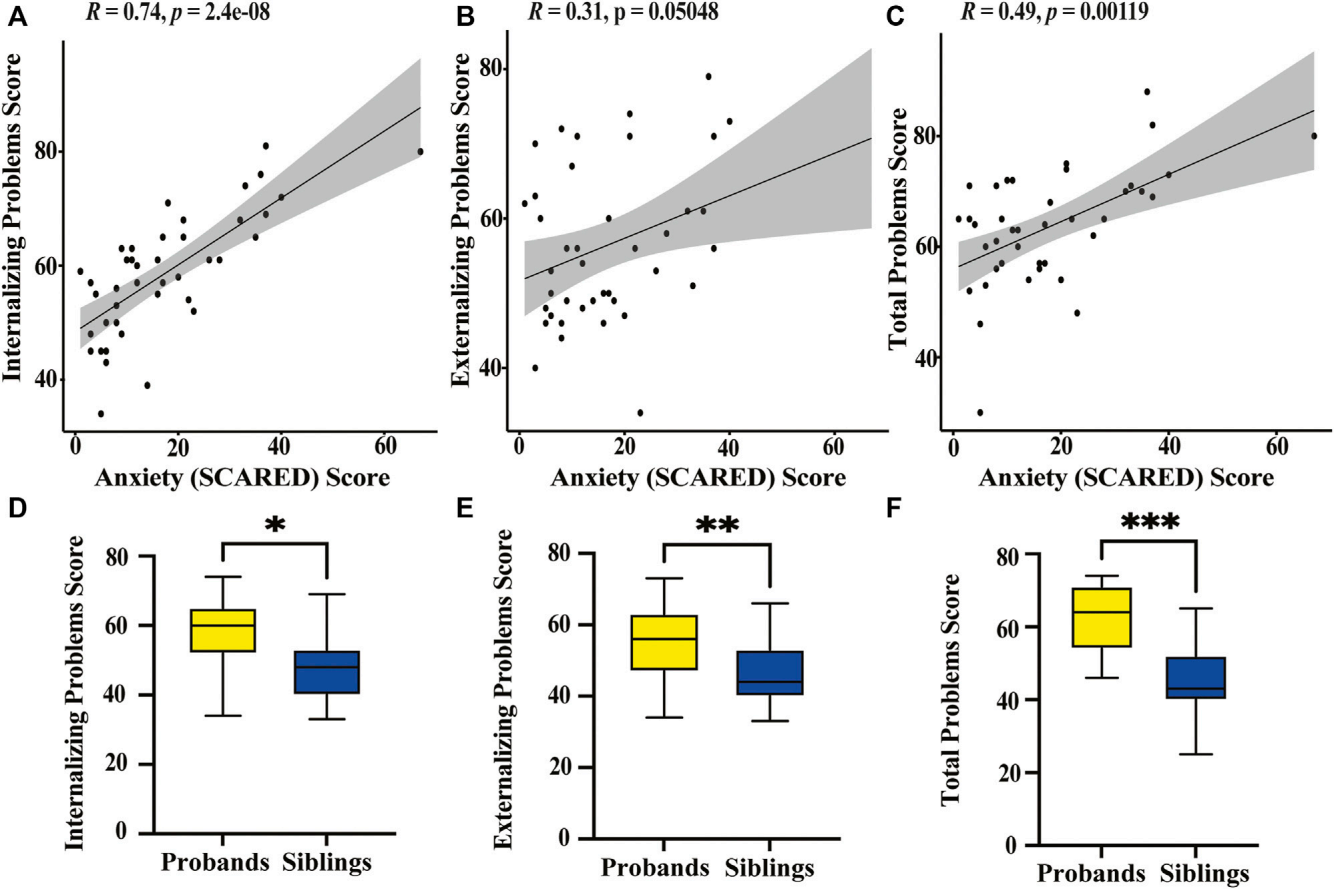
Graphical representation of behavioral survey results **(A)** Spearman’s correlation plot between the SCARED anxiety score for children with KS and the CBCL scores on the internalizing problems category shows a significant positive correlation (*p* < 0.0001). **(B)** Spearman’s correlation plot between the SCARED anxiety scores and the CBCL scores on the externalizing problems category shows no significant correlation (*p* = 0.0505). **(C)** Spearman’s correlation plot between the SCARED anxiety scores and the CBCL total problems category shows a significant positive correlation (*p* = 0.0012). **(D)** Box plot displaying CBCL scores on the internalizing problems category of KS probands (yellow) and their unaffected siblings (blue). *Paired *t*-test reveals a significant difference in scores between sibling pairs (*p* = 0.0009). **(E)** Box plot displaying CBCL scores on the externalizing problems category of KS probands and unaffected siblings. **Paired *t*-test suggests a significant difference between affected and unaffected siblings (*p* = 0.0012). **(F)** Box plot displaying CBCL scores on the total problems category of KS probands and their unaffected siblings. ***Paired *t*-test shows a significant difference in scores between the two groups (*p* < 0.0001).

We find a significant positive correlation between anxiety scores and both internalizing problems (*p* < 0.0001) and total problems (*p* = 0.0012) for children with KS (Figures 3A–C). There is no significant correlation between anxiety scores for adults with KS and any of the behavioral categories. We find there is significant positive correlation between age and internalizing problems (*p* = 0.0104) for children with KS. When performing a paired *t*-test between children with KS and their unaffected siblings for the three broad categories on the CBCL, we find there is a significant difference for internalizing problems, externalizing problems, and total problems, with *p*-values of 0.0009, 0.0012, and <0.0001, respectively (Figures 3D–F). For all other categories, except for anxious/depressed, children with KS scored significantly higher than their unaffected sibling (Table 4).

Averaging scores for all children with KS, only attention problems reached clinical significance as a problem category, with total problems reaching borderline clinical significance. Forty-nine percent of the individual scores of children with KS were in the clinical range for attention problems. Using the DSM-oriented scales, attention deficit for children with KS reached borderline clinically significance, with 37% of individual scores for children with KS in the clinically significant range. The averages of all the other categories fall within the ‘normal’ range. Averaging scores for all adults with KS, only thought problems reached borderline clinically significance, with the averages of all the other categories in the ‘normal’ range. When looking at the percentage of individual scores for all participants with KS that fell in the clinically significant range, the only ones above 20% were thought problems (38.18%), sluggish cognitive tempo (27.27%), and obsessive-compulsive behaviors (25.45%), and attention deficit for children (36.59%) as the ABCL did not have this category. For the unaffected siblings, none of the categories reached clinically significance on the CBCL. There is no significant correlation between attention problems, the only problem category that reached clinical significance, and anxiety scores for children with KS (*p* = 0.5216).

### General cognitive ability and adaptive function in Kabuki syndrome

Individuals with KS showed impairment in all areas of adaptive function on the ABAS-III, with the lowest score in the community use skill area (mean 21.76, SD 18.42). Of the four broad categories (general adaptive composite, social, practical, and conceptual), individuals with KS scored, on average, the highest in the social category, with standard scores ranging from 56 to 115 (mean 81.71, SD 14.02). Within the social category, 20.34% scored extremely low (3 SDs below average), 23.73% scored low (2 SDs below average), 30.51% scored below average (1 SD below average), 22.03% scored average, 3.39% scored above average (1 SD above average), and 0 scored high (2 SDs above average). The general adaptive composite standard scores ranged from 48 to 116 (mean 71.83, SD 15.41). Within the general adaptive composite category, 49.15% scored extremely low, 23.73% scored low, 13.56% scored below average, 10.17% scored average, 3.39% scored above average, and 0 scored high. The conceptual standard scores ranged from 50 to 119 (mean 74.56, SD 15.27). Within the conceptual category, 38.98% scored extremely low, 28.81% scored low, 20.34% scored below average, 10.17% scored average, 1.69% scored above average, and 0 scored high. The practical standard scores ranged from 48 to 112 (mean 68.71, SD 15.80). Within the practical category, 64.41% scored extremely low, 13.56% scored low, 10.17% scored below average, 8.47% scored average, 3.39% scored above average, and 0 scored high.

**FIGURE 4:**
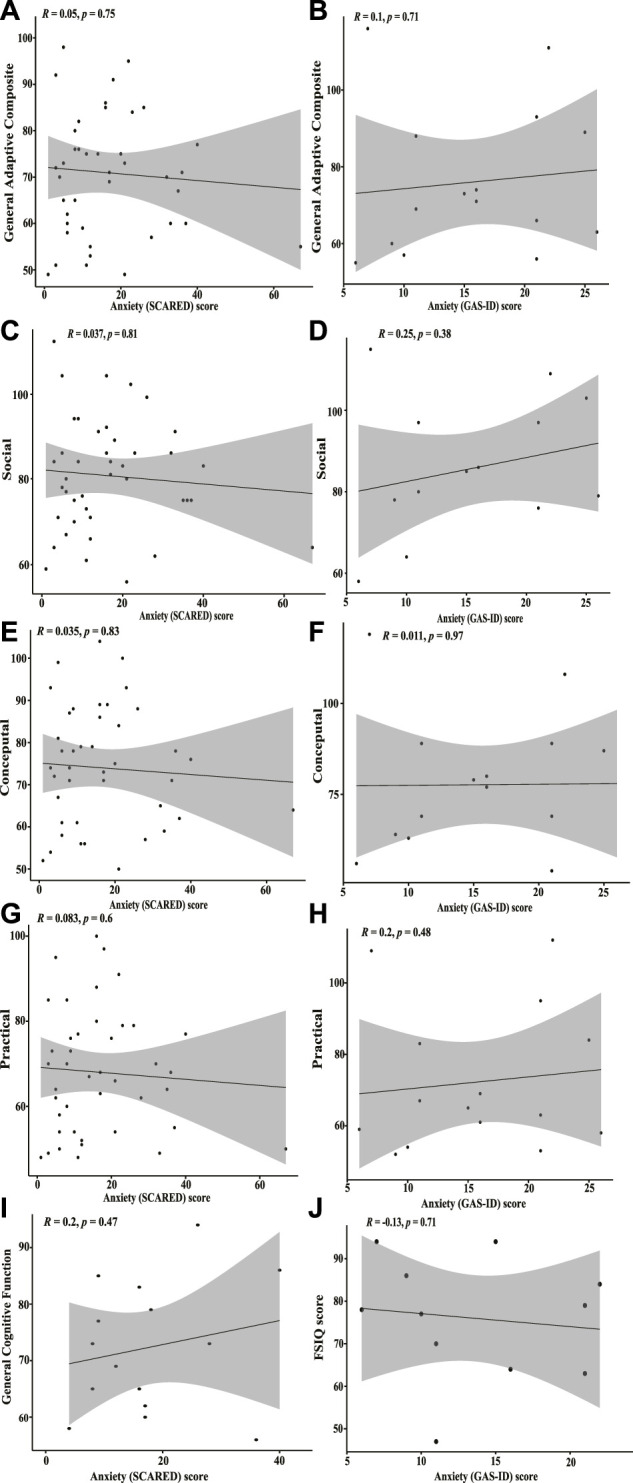
Correlation plots between SCARED/GAS-ID anxiety scores and adaptive function and general cognitive function **(A)** Spearman’s rank correlation plot between SCARED anxiety scores for children with KS and ABAS-III scores from the general adaptive composite category. Correlation is insignificant (*p* = 0.75). **(B)** Pearson’s correlation coefficient plot between GAS-ID anxiety scores for adults with KS and ABAS-III scores from the general adaptive composite category. No significant correlation found (*p* = 0.71). **(C)** Spearman’s rank correlation plot between SCARED anxiety scores and ABAS-III scores from the social category. No significant correlation found (*p* = 0.81). **(D)** Pearson’s correlation coefficient plot between GAS-ID anxiety scores and ABAS-III scores on the social category. Correlation is insignificant (*p* = 0.38). **(E)** Spearman’s rank correlation plot between SCARED anxiety scores and the ABAS-III scores from the conceptual category. No significant correlation found (*p* = 0.83). **(F)** Pearson’s correlation coefficient plot between GAS-ID anxiety scores and the ABAS-III scores from the conceptual category. Correlation is insignificant (*p* = 0.97). **(G)** Spearman’s rank correlation plot between SCARED anxiety scores and the ABAS-III scores from the practical category. No significant correlation found (*p* = 0.6). **(H)** Spearman’s rank correlation plot between GAS-ID anxiety scores and the ABAS-III scores from the practical category. No significant correlation found (*p* = 0.48). **(I)** Spearman’s correlation plot between SCARED anxiety scores and general cognitive function as measured by full-scale intelligence quotient (FSIQ), general intellectual composite, fluid crystalized index, or general ability index. No significant correlation found (*p* = 0.47). **(J)** Pearson’s correlation coefficient plot between GAS-ID anxiety scores and FSIQ scores. Correlation is insignificant (*p* = 0.71).

No significant correlation between anxiety scores and any of the four broad categories was found for either the children nor adult groups with KS (Figures 4A–H). There is no significant correlation between general cognitive function (as measured by historical intelligence testing) and anxiety scores for children with KS (*p* = 0.4744) nor adults with KS (*p* = 0.7142) (Figures 4I,J).

### Positive affect and quality of life

**FIGURE 5:**
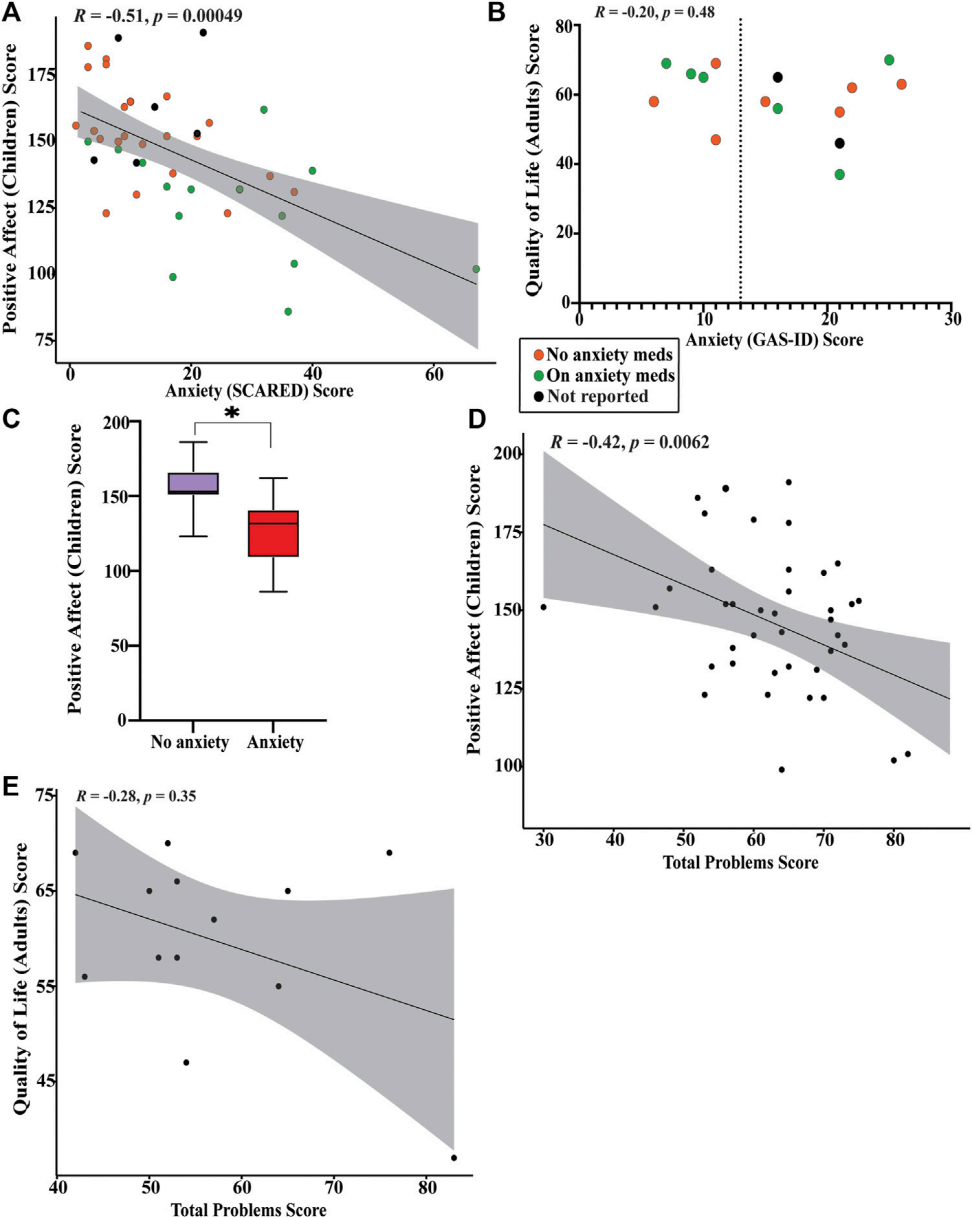
Graphical representation of results from PROMIS-positive affect and quality of life surveys **(A)** Spearman’s rank correlation plot between SCARED anxiety scores and PROMIS-positive affect scores. Those who are actively taking medication for anxiety are shown in green, those that are not are shown in orange, and those that did not respond are shown in black. Significant negative correlation found (*p* = 0.00049). **(B)** Plot of quality of life scores *versus* GAS-ID anxiety scores. Those who are actively taking medication for anxiety are shown in green, those that are not are shown in orange, and those that did not respond are shown in black. No significant correlation found (*p* = 0.48). **(C)** Box plot between children who scored below the SCARED anxiety threshold (purple) and children who scored above the SCARED threshold or scored below the threshold but are currently receiving treatment for anxiety (red). *Significant difference in PROMIS-positive affect scores was found (*p* < 0.0001). **(D)** Pearson’s correlation coefficient plot between CBCL total problems score and PROMIS-positive affect scores. Significant negative correlation found (*p* = 0.0062). **(E)** Spearman’s rank correlation plot between ABCL total problems score and quality of life scores. No significant correlation was found (*p* = 0.35).

The average T-score on the PROMIS positive affect utilized for children with KS is 51.8 (SD 16.1). The average T-score on the adult quality of life measure is 52.3 (SD 15.0). We find there is significant negative correlation between anxiety and positive affect in children with KS (*p* = 0.0005) (Figure 5A) and no significant correlation between anxiety and quality of life in adults with KS (Figure 5B). We find that children with KS that scored below the threshold for anxiety and are not being treated for anxiety have significantly higher positive affect scores than children with KS who surpassed the anxiety threshold or that did not but are receiving treatment for anxiety (*p* < 0.0001) (Figure 5C). There is no significant correlation between increasing number of total behavioral problems and decreasing positive affect for children with KS or decreasing quality of life for adults (Figures 5D,E).

## Discussion

It is known that individuals with intellectual disability experience anxiety at higher rates than the general population, with an increased risk as the individual ages (Reid et al., 2011; Green et al., 2015; Moskowitz et al., 2019). However, our study finds that more than one-third of children and 80% of adults with KS either scored over the threshold for an anxiety disorder and/or are being treated with medication for anxiety, which is significantly higher than what is published in general ID. The rates approach that seen in Fragile X syndrome, a syndrome in which anxiety is known to be a specific phenotypic feature (Edwards et al., 2022). Notably, our numbers are likely even an underestimate as some children and adults who scored below the threshold may be receiving non-pharmacologic treatment for anxiety such as behavioral or psychotherapy. Additionally, the SCARED neglects many symptoms of anxiety in children with neurodevelopmental disorders, such as perseveration and obsessive-compulsive tendencies, and may also miss picking up the drivers of anxiety in this population, such as intolerance to uncertainty. We found that children with KS scored significantly higher on the SCARED than their biological sibling living in the same household, as rated by the same guardian, thus suggesting that the increased anxiety scores are due to KS rather than it being a consequence of other environmental or polygenic factors.

The CBCL and ABCL indicate a rather low rate of behavioral concerns for a population with mild ID. While the children with KS scored significantly higher on almost every behavioral category compared to their siblings, only ADHD fell in the problematic range for children with KS when averaging all CBCL responses. Interestingly, the only category on the CBCL that did not come up as significantly different between children with KS and their unaffected sibling was anxious/depressed. Some reasons for this could be the non-specificity of the category, given that anxiety and depressed are combined, and the tendency for siblings of children with developmental disabilities and profound medical problems to be more depressed. Additionally, when taking the average of all ABCL responses, none of the behavior categories reached clinical significance despite nearly two-thirds of the adults surpassing the threshold for an anxiety disorder on the GAS-ID. Although both the CBCL and ABCL did not indicate anxiety as a clinically significant problem on the DSM-oriented scale, these assessments are not specifically targeted for anxiety and could be missing some of the symptoms of anxiety as it is difficult to tease out anxiety in children as well as in populations with ID. Also, since obsessive-compulsive symptoms are separated out, this may account for the discrepancy. In fact, when looking at individual subscale scores on the CBCL/ABCL, the only ones where greater than 20% of KS participants were in the clinically significant range were attention problems, thought problems, sluggish cognitive tempo, obsessive-compulsive behaviors, and attention deficit problems which all highly relate to either ADHD or anxiety. Since the SCARED and GAS-ID specifically focus on anxiety screening, we relied more on those assessments to measure anxiety, although as mentioned before, even these are inadequate and further research should be done.

Another reason we feel anxiety is clinically meaningful in this population is because only anxiety and not total behavioral problems nor attention problems was negatively correlated with positive affect in children with KS, indicating that anxiety is a major issue that is influencing positive affect, despite other behaviors coming up as clinically significant problems. While anxiety did not correlate with decreasing quality of life in adults with KS, 80% of the adults are scoring either above the threshold for anxiety or below the threshold but on anxiety medication, so it is difficult to make conclusions about correlations with such a small number not having anxiety.

Lastly, the anxiety data did not correlate with the adaptive function of the affected children nor adults, nor did it correlate with overall cognitive ability for either group, suggesting that the anxiety seen is not simply correlated with more impaired function. Together, our findings suggest that anxiety is a prevalent phenotypic feature of KS that cannot be solely or adequately explained by the presence of intellectual disability, nor due to environmental and familial polygenic factors, and is impactful on positive affect.

Mouse models of KS demonstrate impaired hippocampal neurogenesis and smaller dentate gyri, as well as problems with visuospatial memory as indicated by the Morris water maze performance (Bjornsson et al., 2014). Furthermore, individuals with KS demonstrate cognitive profiles consistent with dysfunction of the dentate gyrus (Harris et al., 2019). It is known that SSRIs, which are commonly used to treat anxiety disorders, stimulate neurogenesis in the hippocampus (Cassano et al., 2002; Taupin, 2006). As such, it makes biological sense that anxiety would be a feature of KS and this study further supports this hypothesis.

Our conclusions from this study are informative to clinicians caring for individuals with KS as they emphasize the need for anxiety screening in all individuals with KS. Additionally, this study demonstrates that despite individuals with KS scoring highly on measures of positive affect and overall quality of life, the anxiety scores of children with KS are strongly correlated with decreasing positive affect, which underscores the importance of looking for and treating the anxiety. As the children who scored below the threshold for anxiety have significantly higher positive affect scores than children who scored above the threshold or scored below but are currently being treated for anxiety, it is important for clinicians to adequately treat the anxiety, as well as ensure that the treatment is working long term. Treatment should include behavioral therapy interventions, IEP modifications, and pharmacology as appropriate. Since individuals with KS were shown not to display many clinically significant behavioral issues as per the CBCL/ABCL results, clinicians should carefully screen for anxiety, as well as ADHD, which was found to be a clinically significant problem. In addition to clinical ramifications, it is also important to establish anxiety as a phenotype of KS because this phenotype may be a proxy for hippocampal neurogenesis in these patients (Kheirbek and Hen, 2014) and can potentially be used as an outcome measure in future clinical trials.

Our study has several limitations that should be addressed in future research. First, the SCARED is used frequently in research, however, it can miss anxiety manifesting in a child with neurodevelopmental disabilities (i.e. the need for routine, perseveration, obsessive-compulsive symptoms). Also, since we used parent reported surveys for the children, it is possible that the accuracy of collected data is biased, given that anxiety is an internal and personal experience (Stern et al., 2014). Another limitation is that while the sibling group was used as a way to account for environmental and polygenic factors, we did not use a comparison group of idiopathic ID or another genetic syndrome with ID. Instead, published historical data was used to estimate anxiety prevalence in other syndromes. In the future, a KS group could be directly compared to a group with a different genetic cause of ID in order to determine if anxiety is a specific feature of KS. Our data is also skewed more towards younger individuals as we had more child participants and the mean age of the adult group can be considered younger adulthood. The molecular basis of KS was not reported until 2010, despite the syndrome first being described clinically in 1981 (Kuroki et al., 1981; Niikawa et al., 1981; Ng et al., 2010). As we required molecular confirmation for participation in this study, most interested participants were of younger age as there are most likely many adults with KS that are undiagnosed or do not have molecular confirmation. Since anxiety and risk factors change over an individual’s lifespan, it is possible the data is not as generalizable to older individuals. This study also focused primarily on anxiety but the data indicates signal in other types of problems in children with KS, particularly ADHD, and incomplete assessments of these other behaviors may lead us to overweigh the impact of anxiety. Lastly, measures to fully look at overall quality of life in children and adults should be used to assess the full impact of anxiety and other factors on individuals with KS.

Future studies could build on this study and use more complete measures of both anxiety and quality of life that are more comparable across adults and children and include patient and caregiver reported measures as well as clinician-rated measures to better design outcome measures and improve clinical care. Future studies could also look more in depth at other behavioral problems including ADHD. What is more, while it is interesting that there is significant positive correlation between anxiety scores and age in children and that a larger proportion of adults than children are scoring over the threshold for anxiety in this study, this is a cross-sectional study that does not detect change over time, so a longitudinal study would be needed to further understand the impact of aging on anxiety in KS. Additionally, larger numbers of patients could allow for better genotype-phenotype correlation.

In conclusion, this study has shown that anxiety is a prominent part of the neurobehavioral phenotype of KS that should be carefully screened for in all age groups. In addition to improving the clinical care of those with KS, this study also contributes to the design of future clinical trials researching potential treatments for KS, as reduced anxiety levels could act as a measurable and clinically-relevant outcome measure in the future.

## Data Availability

The original contributions presented in the study are included in the article/Supplementary Material, further inquiries can be directed to the corresponding author.
