# Supplementary material for: Neurobehavioral phenotype of Kabuki syndrome: Anxiety is a common feature
Source: Front Genet. 2022 Oct 6;13:1007046. doi: 10.3389/fgene.2022.1007046 (PMC9582441; doi:10.3389/fgene.2022.1007046)
Supplement: Supplementary file 1 [file DataSheet1.docx]

**Supplementary Table 1**

| **ID** | **Age** | **Sex** | **Variant** | **Mutation Type (P, L, V)** | **General Cognitive Function** | **Anxiety Score** | **Anxiety Meds (Y/N)** | **CBCL/**  **ABCL Total Problems Score** | **ABAS**  **(GAC)**  **Score** | **Positive Affect/ QOL**  **Score** | **Sibling (Y/N)** | **Sibling Age** | **Sibling Sex** | **Sibling Anxiety Score** | **Sibling CBCL Total Problems** |
| --- | --- | --- | --- | --- | --- | --- | --- | --- | --- | --- | --- | --- | --- | --- | --- |
| KS0112 | 23 | F | KMT2D  c.7903C>T | Nonsense (P) | 79 | 21/54 | Y | 83-C | 66 | 37/76 | N |  |  |  |  |
| KS0221 | 10 | M | KMT2D  c.15535C>T | Missense (P) | 73 | 28/82 | Y | 65-C | 57 | 132/195 | Y | 6 | F | 13/82 | 45 |
| KS0322 | 22 | M | KMT2D  c.5124_5125delAC | Frameshift deletion (P) | 56 | 36/82 | Y | 88-C | 71 | 86/195 | N |  |  |  |  |
| KS0412 | 35 | F | KMT2D  c.14580dupT | Frameshift insertion (P) | 94 | 15/54 | N | 53 | 73 | 58/76 | N |  |  |  |  |
| KS0522 | 15 | M | KMT2D  c.3190dupG | Frameshift insertion (P) | 62 | 17/82 | N | 57 | 71 | 138/195 | N |  |  |  |  |
| KS0622 | 19 | M | KMT2D  c.15143G>A | Missense (L) | 69 | 12/82 | Y | 60-B | 53 | 142/195 | N |  |  |  |  |
| KS0722 | 11 | F | KMT2D  c.5278_5279delAA | Frameshift deletion (P) | 79 | 18/82 | Y | 68-C | 91 | 122/195 | Y | 8 | M | 13/82 | 45 |
| KS0912 | 19 | F | KMT2D  c.7481_7482insT | Frameshift insertion (P) | 63 | 21/54 | N | 64-C | 56 | 55/76 | N |  |  |  |  |
| KS1022 | 12 | F | KMT2D  c.11263C>T | Nonsense (P) | 77 | 9/82 | N | 57 | 76 | 152/195 | N |  |  |  |  |
| KS1121 | 16 | F | KMT2D  c.7291_7294delTCTG | Frameshift deletion (P) | NR | 8/82 | NR | 56 | 65 | 189/195 | Y | 15 | F | 5/82 | 52 |
| KS1312 | 29 | F | KMT2D  c.15536G>A | Missense (P) | 77 | 10/54 | Y | 65-C | 57 | 65/76 | N |  |  |  |  |
| KS1422 | 14 | F | KMT2D  IVS50+5G>A | Splice site (L) | 83 | 16/82 | N | 56 | 85 | 152/195 | N |  |  |  |  |
| KS1522 | 15 | M | KMT2D  c.16501C>T | Nonsense (P) | 65 | 16/82 | Y | 57 | 86 | 133/195 | N |  |  |  |  |
| KS1722 | 14 | F | KMT2D  c.16437delT | Frameshift deletion (P) | 73 | 8/82 | N | 61-B | 80 | 150/195 | N |  |  |  |  |
| KS1821 | 5 | M | KMT2D  c.4209C>A | Nonsense (P) | TY | 16/82 | N | NC | 102 | 167/195 | Y | NR | NR | 14/82 | NR |
| KS1922 | 16 | M | KMT2D  c.10369_10370delCT | Frameshift deletion (P) | NR | 37/82 | N | 69-C | 60 | 131/195 | N |  |  |  |  |
| KS2022 | 19 | F | KDM6A  c.3144+1delG | Splice Site (L) | NR | 67/82 | Y | 80-C | 55 | 102/195 | N |  |  |  |  |
| KS2121 | 5 | M | KMT2D  c.175A>G | Missense (P) | TY | 5/82 | N | 46 | 98 | 151/195 | Y | 8 | M | 10/82 | 31 |
| KS2222 | 16 | M | KMT2D  c.4343G>A | Missense (L) | NR | 37/82 | Y | 82-C | NC | 104/195 | N |  |  |  |  |
| KS2312 | 21 | F | KMT2D  c.10813C>T | Nonsense (P) | 78 | 6/54 | N | 51 | 55 | 58/76 | N |  |  |  |  |
| KS2421 | 5 | F | KMT2D  c.12844C>T | Nonsense (P) | TY | 1/82 | N | 65-C | 49 | 156/195 | Y | 10 | F | 14/82 | 41 |
| KS2522 | 9 | M | KMT2D  c.4421G>T | Missense (L) | 85 | 9/82 | N | 65-C | 82 | 163/195 | N |  |  |  |  |
| KS2612 | 28 | M | KMT2D  c.14006C>G | Nonsense (P) | 64 | 16/54 | Y | 43 | 74 | 56/76 | N |  |  |  |  |
| KS2721 | 20 | M | KMT2D  c.12592C>T | Nonsense (P) | 86 | 9/54 | Y | 53 | 60 | 66/76 | N |  |  |  |  |
| KS2821 | 7 | F | KDM6A  c.2362-2372dup11 | Frameshift (P) | NR | 21/82 | N | 74-C | 49 | 152/195 | Y | 12 | M | 2/82 | 27 |
| KS2921 | 17 | F | KMT2D  c.2578_2579del | Frameshift deletion (P) | NR | 3/82 | Y | 71-C | 51 | 150/195 | N |  |  |  |  |
| KS3012 | 23 | F | KMT2D  c.9602dupT | Frameshift insertion (P) | NR | 25/54 | Y | 52 | 89 | 70/76 | N |  |  |  |  |
| KS3122 | 8 | M | KMT2D  c.2533delC | Frameshift deletion (P) | NR | 35/82 | Y | 70-C | 67 | 122/195 | N |  |  |  |  |
| KS3221 | 13 | M | KMT2D  c.12592C>T | Nonsense (P) | NR | 4/82 | NR | 64-C | 70 | 143/195 | Y | 16 | F | 21/82 | 65-C |
| KS3321 | 9 | F | KMT2D  c.9144C>G | Missense (V) | 86 | 40/82 | Y | 73-C | 77 | 139/195 | Y | 7 | M | 17/82 | 48 |
| KS3421 | 5 | F | KDM6A  c.357C>G | Nonsense (P) | TY | 6/82 | N | 53 | 58 | 181/195 | Y | 3 | F | 1/82 | 25 |
| KS3622 | 7 | F | KMT2D | Nonsense (P) | NR | 11/82 | NR | 72-C | 51 | 142/195 | Y | 11 | M | 8/82 | 43 |
| KS3721 | 5 | M | KMT2D  c.14194C>T | Nonsense (P) | TY | 4/82 | N | NC | NC | 154/195 | N |  |  |  |  |
| KS3812 | 18 | F | KMT2D  c.4265G>A | Nonsense (P) | 70 | 11/54 | N | 42 | 88 | 69/76 | N |  |  |  |  |
| KS3921 | 7 | F | KMT2D  c.15289 C>T | Nonsense (P) | NR | 20/82 | NR | 54 | 75 | 132/195 | Y | 9 | F | 6/82 | 42 |
| KS4012 | 27 | F | KMT2D  c.15536G>A | Missense (L) | 94 | 7/54 | Y | 76-C | 116 | 69/76 | N |  |  |  |  |
| KS4122 | 13 | M | KMT2D  c.15884G>C | Missense (L) | NR | 3/82 | N | 52 | 92 | 186/195 | Y | 10 | M | 3/82 | 40 |
| KS4222 | 5 | M | KMT2D  c.6180C>A | Nonsense (P) | TY | 5/82 | N | NC | 65 | NC | N |  |  |  |  |
| KS4322 | 6 | F | KMT2D  c.2579delT | Frameshift deletion (P) | TY | 6/82 | N | 60-B | 60 | 179/195 | N |  |  |  |  |
| KS4621 | 10 | F | KDM6A  deletion of exons 3 and 4 | Frameshift deletion (P) | 65 | 8/82 | Y | 71-C | 76 | 147/195 | Y | 14 | M | 8/82 | 64-C |
| KS4721 | 5 | M | KMT2D  c.2164delG | Frameshift deletion (P) | TY | 6/82 | N | 53 | 62 | 123/195 | N |  |  |  |  |
| KS4812 | 19 | F | KDM6A  c.4087C>T | Nonsense (P) | 47 | 11/54 | N | 54 | 69 | 47/76 | N |  |  |  |  |
| KS5221 | 8 | F | KMT2D  c.15559G>C | Missense (L) | 94 | 26/82 | N | 62-B | 85 | 123/195 | Y | 5 | F | 15/82 | 54 |
| KS5312 | 30 | M | KMT2D  c.2819N>G | Nonsense (P) | NR | 26/54 | N | NC | 63 | 63/76 | N |  |  |  |  |
| KS5422 | 16 | M | KMT2D  c.2854delT | Frameshift deletion (L) | 60 | 17/82 | Y | 64-C | 69 | 99/195 | Y | 20 | M | 2/82 | 41 |
| KS5512 | 20 | M | KMT2D  c.13432dupC | Frameshift insertion (P) | 84 | 22/54 | N | 57 | 111 | 62/76 | N |  |  |  |  |
| KS5622 | 6 | F | KDM6A c.4005+5_4005+8delGTAA | Intronic, likely splice site (P) | TY | 23/82 | N | 48 | 84 | 157/195 | Y | 4 | M | 4/82 | 36 |
| KS5721 | 13 | M | KMT2D  c.12133C>T | Nonsense (P) | NR | 11/82 | N | 63-B | 75 | 130/195 | Y | 11 | M | 0/82 | 40 |
| KS5912 | 22 | M | KMT2D  c.15943C>T | Nonsense (P) | NR | 16/54 | NR | 50 | 71 | 65/76 | N |  |  |  |  |
| KS6121 | 5 | M | KMT2D c.3900_3906+1delCAAACAGGinsGAAACAGT | Splice site* (P) | TY | 12/82 | N | 63-B | 55 | 149/195 | Y | 15 | F | 15/82 | 29 |
| KS6322 | 5 | M | KMT2D  c.15800T>A | Missense (L) | TY | 3/82 | N | 65-C | 72 | 178/195 | Y | 10 | M | 10/82 | 55 |
| KS6422 | 8 | M | KMT2D  c.6295C>T | Nonsense (P) | NR | 22/82 | NR | 65-C | 95 | 191/195 | N |  |  |  |  |
| KS6622 | 8 | F | KDM6A  Xp.11.4p11.3 deletion | Intragenic deletion (P) | NR | 21/82 | NR | 75-C | 73 | 0.7846 | N |  |  |  |  |
| KS6822 | 4 | M | KMT2D  c.8045_8046delAG | Frameshift deletion (P) | TY | 10/82 | N | 72-C | 59 | 165/195 | Y | 13 | F | 18/82 | 48 |
| KS6921 | 11 | F | KMT2D  c.15922-2A>G | Intronic splice site (P) | NR | 33/82 | N | 71-C | 60 | 137/195 | Y | 8 | M | 16/82 | 63-B |
| KS7121 | 7 | F | KMT2D  c.3334delC | Frameshift deletion (P) | NR | 14/82 | NR | 54 | 75 | 163/195 | Y | 9 | F | 8/82 | 47 |
| KS7222 | 6 | M | KMT2D  c.16501C>T | Nonsense (P) | TY | 5/82 | N | 30 | 73 | 151/195 | N |  |  |  |  |
| KS7322 | 11 | M | KMT2D  c.10966C>T | Missense (V) | NR | 23/82 | Y | NC | NC | NC | Y | 16 | F | 26/82 | NC |
| KS7412 | 43 | F | KMT2D  c.10966C>T | Missense (V) | NR | 21/54 | NR | NC | 93 | 46/76 | N |  |  |  |  |
| KS7522 | 11 | F | KMT2D  c.16018C>T | Nonsense (P) | NR | 32/84 | Y | 70-C | 70 | 162/195 | Y | 10 | M | 8/82 | 42 |

Mutation Type (P, L, V), Pathogenic, Likely pathogenic, Variant of unknown significance confirmed with Episign; CBCL, Child Behavior Checklist; ABCL, Adult Behavior Checklist; ABAS, Adaptive Behavior Assessment System; GAC, General adaptive composite, QOL, Quality of life.

TY = too young, NR = not reported, NC= not completed.

*KS6121 variant was classified as an indel by the calling algorithm. However, upon careful examination of the variant it appears to be two single-base changes, one being a splice site and the other being a synonymous exotic base pair change.

For CBCL/ABCL Total problems column, #-B = borderline clinically significant and #-C = clinically significant.

**Supplementary Table 2**

| **SCARED- Anxiety type** | **Cutoff value** | **Number of KS probands scoring above threshold** | **Number of siblings scoring above threshold** |
| --- | --- | --- | --- |
| ***Panic disorder or significant somatic symptoms*** | >=7 | 7 | 0 |
| ***Generalized anxiety disorder*** | >=9 | 8 | 1 |
| ***Separation anxiety*** | >=5 | 13 | 6 |
| ***Social Anxiety*** | >=8 | 10 | 0 |
| ***Significant school avoidance*** | >=3 | 9 | 0 |

SCARED, Screen for Child Anxiety Related Emotional Disorders.
